# SOCS3 Attenuates GM-CSF/IFN-γ-Mediated Inflammation During Spontaneous Spinal Cord Regeneration

**DOI:** 10.1007/s12264-020-00493-8

**Published:** 2020-04-18

**Authors:** Xuejie Zhang, Bingqiang He, Hui Li, Yingjie Wang, Yue Zhou, Wenjuan Wang, Tiancheng Song, Nan Du, Xingxing Gu, Yi Luo, Yongjun Wang

**Affiliations:** 1 Key laboratory of Neuroregeneration of Jiangsu and The Ministry of Education, Nantong University, Nantong, 226001 China; 2 Co-innovation Center of Neuroregeneration, Nantong University, Nantong, 226001 China; 3 Department of Rehabilitation Medicine, Affiliated Hospital of Nantong University, Nantong, 226001 China

**Keywords:** SOCS3, Spinal cord, Vertebrate, Inflammation, Cytokine

## Abstract

SOCS3, a feedback inhibitor of the JAK/STAT signal pathway, negatively regulates axonal regrowth and inflammation in the central nervous system (CNS). Here, we demonstrated a distinct role of SOCS3 in the injured spinal cord of the gecko following tail amputation. Severing the gecko spinal cord did not evoke an inflammatory cascade except for an injury-stimulated elevation of the granulocyte/macrophage colony-stimulating factor (GM-CSF) and interferon gamma (IFN-γ) cytokines. Simultaneously, the expression of SOCS3 was upregulated in microglia, and unexpectedly not in neurons. Enforced expression of SOCS3 was sufficient to suppress the GM-CSF/IFN-γ-driven inflammatory responses through its KIR domain by attenuating the activities of JAK1 and JAK2. SOCS3 was also linked to GM-CSF/IFN-γ-induced cross-tolerance. Transfection of adenovirus overexpressing SOCS3 in the injured cord resulted in a significant decrease of inflammatory cytokines. These results reveal a distinct role of SOCS3 in the regenerating spinal cord, and provide new hints for CNS repair in mammals.

**Electronic supplementary material:**

The online version of this article (10.1007/s12264-020-00493-8) contains supplementary material, which is available to authorized users.

## Introduction

Traumatic spinal cord injury (SCI) always results in an excessive inflammatory response, which contributes to secondary tissue damage that is characterized by the formation of an ellipsoidal, loculated cystic cavity [1–4]. Inflammation is initiated by infiltrating leukocytes or resident microglia that bind with endogenous damage-associated molecular pattern (DAMP) molecules through their pattern recognition receptors on the cell surface [5]. The subsequent release of cytokines and chemokines from these cells in turn recruits and activates more immune cells, forming a progressive inflammatory cascade at the site of tissue damage. The deteriorating milieu is neurotoxic, with tumor necrosis factor alpha (TNF-α), interleukin (IL)-1β, glutamate, nitric oxide synthase (iNOS), and free radicals [6, 7]. Inflammation starts soon after SCI and is sustained for days and weeks. Pharmaceutical treatment, such as methylprednisone, has been shown to delay post-traumatic inflammation and retard neuronal degeneration in the central nervous system (CNS) [8, 9].

Granulocyte/macrophage colony-stimulating factor (GM-CSF) was first characterized as a soluble factor with the ability to differentiate bone marrow precursor cells into granulocytes and macrophages [10, 11]. Later, it was proposed to support myeloid cell survival and proliferation in a dose-dependent manner [10, 12], and its pathophysiological significance has been emphasized in its association with inflammation and autoimmune diseases. Evidence indicates that the aberrant expression of GM-CSF exacerbates autoimmune diseases such as rheumatoid arthritis, which is a chronic inflammatory disorder characterized by joint pain and deterioration [13], and experimental autoimmune encephalomyelitis, which is an autoimmune disorder of the CNS [14, 15]. Macrophages are one of the GM-CSF-sensitive cell types that release inflammatory cytokines, including IL-1, TNF-α, and IL-6 [16, 17]. In the CNS, GM-CSF-driven inflammation by macrophages/microglia seems more relevant to tissue damage and pathogenicity than that of T-cells, as the latter do not express the GM-CSF receptor α-chain [11, 18, 19]. The receptor for GM-CSF is abundant on macrophages and other myeloid cells. Ligation of GM-CSF to the receptor activates intracellular signaling by the trans-phosphorylation of Janus kinase 2 (JAK2), which triggers the activation of downstream STAT5 and associated kinases [20–22]. IFN-γ, the priming stimulus for macrophages, can act synergistically with GM-CSF to promote the production of cytokines [17, 23]. The signaling displays a crosslink with GM-CSF, as the α and β chains of the IFN-γ receptor are constitutively associated with JAK1 and JAK2, respectively [23].

Suppressor of cytokine signaling-3 (SOCS3) is a feedback inhibitor of the JAK/STAT signaling pathway [24, 25]. Several cytokines activate the JAK/STAT pathway by binding to their associated cell-surface receptors, leading to the recruitment and phosphorylation of receptor-associated JAK1 and JAK2, which phosphorylate and cause dimerization of the STAT proteins. These proteins promote the expression of target genes including SOCS3, which in turn negatively regulates JAK/STAT signaling by binding to the receptors or JAKs *via* a 12-amino-acid kinase-inhibitory region (KIR) [24, 26]. SOCS3 has been found to suppress inflammation in response to multiple stimuli in the contexts of pathophysiology and tissue damage [24, 25, 27, 28]. In addition, SOCS3 is rapidly induced in neurons with trauma of the peripheral and central nervous systems, and it plays inhibitory roles in neuronal regrowth. Its deficiency in primary sensory neurons or adult retinal ganglion cells results in a significant promotion of axonal elongation [26, 29]. The negative regulation of SOCS3 in both inflammatory responses and axonal growth leads to a somewhat difficult tradeoff in promoting CNS repair without evoking inflammation.

Unlike mammals, regenerating organisms such as fish, amphibians, and reptiles are capable of spinal cord regeneration after injury [30–35]. Clearly, axonal regrowth in these non-mammals occurs in a permissive milieu without inhibitory extracellular matrix components, glial scarring, or excessive activation of inflammation [30, 32]. Some DAMPs and cytokines are released but do not evoke uncontrolled inflammatory cascades [31]. A real possibility is that some negative regulators of inflammation play active roles in the regenerative process, but the details of such regulatory mechanisms remain unclear. Since the signal transduction of SOCS3, GM-CSF, and IFN-γ converge on JAK/STAT signaling, we used *Gekko japonicus* as an experimental SCI model to investigate the regulatory mechanism of SOCS3 in suppressing inflammation that was boosted by GM-CSF and IFN-γ without affecting axonal regrowth during natural spinal cord regeneration.

## Materials and Methods

### Animals

Adult *G. japonicus* were used as described by Dong *et al*. [31]. Briefly, adult geckos were fed *ad libitum* with mealworms and housed in an air-conditioned room with a controlled temperature (22 °C–25 °C) and saturated humidity. Anesthesia was induced by cooling the geckos on ice prior to tail amputation. Amputation was performed at the sixth caudal vertebra, which was identified based on the special tissue structure that is present at that position [36], by placing a slipknot of nylon thread and pulling gently until the tail was detached, thus mimicking the natural defense mechanism.

All of the experiments were conducted in accordance with the guidelines of the NIH (*Guide for the Care and Use of Laboratory Animals*, 1985) and the Society for Neuroscience (*Guidelines for the Use of Animals in Neuroscience Research*). The experiments were approved by the Animal Care and Use Committee of Nantong University and the Jiangsu Province Animal Care Ethics Committee. All geckos were anesthetized on ice prior to sacrifice.

### Cloning and Analysis of Gecko SOCS3

To obtain the full length of gecko SOCS3, the anti-sense primer 5′–TAG AAG AGG CGG TGG TGG CA–3′ and the sense primer 5′–ACA CCT TTC CTC CCC TGC CG–3′ were designed according to their genome sequences [33]. Both 5′–rapid amplification of cDNA ends (RACE) and 3′–RACE were performed using the BD SMART RACE cDNA amplification kit (Clontech, USA) according to the manufacturer’s instructions. Comparison against the GenBank protein database was performed using the PSI-BLAST network server at the National Center for Biotechnology Information [37]. Multiple protein sequences were aligned by the MegAlign program using the Clustal method in the DNASTAR software package [38].

### Production of Adenovirus Overexpressing SOCS3

Adenoviruses (GV314-SOCS3 or GV314-mSOCS3) overexpressing SOCS3 or mutated SOCS3 (F4A) were produced by Genechem Co. Ltd. (Shanghai) according to the manufacturer’s procedures. The open reading frame of SOCS3 family members was cloned into a GV314 vector *via* the Bam HI and AgeI sites. SOCS3 expression was driven by the EF-1α promoter, and the expression of the reporter-enhanced green fluorescent protein (eGFP) was driven by a CMV promoter. Both SOCS3 and the eGFP sequence were incorporated into an adenovirus. The adenovirus was produced using 293T cells, and the viral titers reached 2 × 10^10^ plaque-forming units/mL for further studies.

### Cell Culture and Treatment

Mouse macrophage RAW 264.7 cells (Chinese Academy of Sciences, Shanghai Institutes for Biological Sciences Cell Resource Center) were cultured in Dulbecco’s modified Eagle’s medium (Gibco BRL) supplemented with 10% (*v*/*v*) fetal bovine serum in a 37 °C humidified incubator with 5% CO_2_. The cells were treated with 10 ng/mL IFN-γ and/or 50 ng/mL GM-CSF for 0.5 h–2 h (R&D Systems) or transfected with a GV314-vector, GV314-SOCS3 or a GV314-mSOCS3 adenovirus for 48 h before stimulation with the cytokines, and then collected for subsequent assays.

### Quantitative Real-Time Polymerase Chain Reaction (Q-PCR)

Total RNA was prepared with TRIzol (Gibco) from mouse macrophage RAW 264.7 cells after the designated treatment. For the Q-PCR examination of cytokine transcriptional expression, first-strand cDNA was synthesized using the Omniscript reverse transcription kit (Qiagen) in a 20-µL reaction system containing 2 µg total RNA, 0.2 U/µL M-MLV reverse transcriptase, 0.5 mmol/L dNTP mix, and 1 µmol/L Oligo-dT primer. The cDNA was diluted 1:4 before use in Q-PCR assays. The sequence-specific primers were designed and synthesized by Invitrogen (Shanghai, China). The primer pair and probe for IL-6 were: forward primer 5′–GCG AGA GTC CTT CAG AGA GA–3′, reverse primer 5′–GGT CTT GGT CCT TAG CCA CT–3′; and for iNOS were: forward primer 5′–TGT GCT CCA TAG TTT CCA GAA G–3′, reverse primer 5′–GGA CAT AGT TCA ACA TCT CCT GG–3′. The Q-PCR reactions were performed in a final volume of 20 μL (1 μL cDNA template and 19 μL Q-PCR reaction buffer containing 2.5 mmol/L MgCl_2_, 0.2 mmol/L dNTPs, 0.5 μmol/L anti-sense and sense primers, 0.4 μmol/L TaqMan probe, 0.2 μL DNA polymerase, and 1×DNA polymerase buffer). Rotor-Gene 5 software (Rotor-Gene, Corbett Research, Sydney, Australia) was used for the real-time PCR analysis. The reactions were processed using one initial denaturation cycle at 94 °C for 5 min followed by 45 cycles of 94 °C for 30 s, 60 °C for 30 s, and 72 °C for 30 s. Fluorescence was recorded during each annealing step. At the end of each PCR run, the data were automatically analyzed by the system, and the amplification plots were obtained. The full-length SOCS3 plasmid was used to prepare the standard curves and was used as a specificity control for real-time PCR. The expression levels of the cytokines were normalized to an endogenous GAPDH cDNA using the forward primer 5′–TGA AGT CGC AGG AGA CAA CC–3′ and the reverse primer 5′–GGT GGA GCC AAA AGG GTC A–3′. In addition, a negative control without the first-strand cDNA was included.

### ELISA Assays

A two-site sandwich ELISA was used to quantify TNF-α and IL-1β in the extracts of 0.5-cm spinal cord segments following GV314-SOCS3 adenovirus transfection, or in the extracts of RAW 264.7 cells cultured with 10 ng/mL IFN-γ and/or 50 ng/mL GM-CSF recombinant proteins for 2 h following adenovirus transfection for 48 h. The cell supernatants were harvested and the cells were lysed in buffer containing 1% SDS, 100 mmol/L Tris-HCl, 1 mmol/L PMSF, and 0.1 mmol/L β-mercaptoethanol. The lysates were centrifuged at 12,000 g for 15 min. The levels of TNF-α and IL-1β were assessed using the appropriate ELISA kits (BD Biosciences) according to the manufacturer’s directions. The plates were read using a 96-well plate reader (Biotek Synergy2) at a wavelength of 450 nm.

### Western Blot

Proteins were extracted from 0.5-cm spinal cord segments or cultured cells with a buffer containing 1% SDS, 100 mmol/L Tris–HCl, 1 mmol/L PMSF, and 0.1 mmol/L β-mercaptoethanol. The protein concentration of each specimen was assessed by the Bradford method to maintain the same loads. The protein extracts were heat-denatured at 95 °C for 5 min, electrophoretically separated on 10% SDS–PAGE, and transferred to PVDF membranes. The membranes were reacted with a 1:1000 dilution of primary antibodies in TBS buffer at 4 °C overnight, followed by reaction with a secondary antibody conjugated with goat anti-rabbit or goat anti-mouse HRP (Proteintech), diluted 1:1000, at room temperature for 2 h. After the membrane was washed, the HRP activity was detected using an ECL kit. The image was scanned with a GS800 densitometer scanner (Bio-Rad), and the data were analyzed using ImageJ 1.41o (National Institutes of Health). β-actin (1:5000) was used as an internal control. The antibodies used in the Western blots were: SOCS1 or SOCS3 (polyclonal rabbit anti-gecko antibody commercially prepared using peptides synthesized by GenScript Biotech Corp., or purchased from Abcam), STAT1 (Cell Signaling Technology), pSTAT1 Tyr701 (Abcam), STAT3 (Cell Signaling Technology), pSTAT3 Tyr705 (Cell Signaling Technology), STAT5 (Cell Signaling Technology), pSTAT5 Tyr694 (Cell Signaling Technology), JAK1 (Merck), JAK2 (Cell Signaling Technology), and β-actin (Proteintech).

### Immunoprecipitation

The macrophage RAW 264.7 cells were washed twice with cold phosphate-buffered saline and then extracted with lysis buffer (20 mmol/L Tris-HCl, pH 7.5, 150 mmol/L NaCl, 1 mmol/L EDTA, 1 mmol/L EGTA, 1% Triton X-100, 2.5 mmol/L sodium pyrophosphate, 1 mmol/L β-glycerophosphate, 1 mmol/L Na_3_VO_4_, 1 mmol/L phenylmethylsulphonyl fluoride, and Roche Applied Science’s complete protease inhibitors). Whole cell extracts were centrifuged at 14,000 rpm for 20 min to remove the debris. The proteins in the supernatant were measured using Protein Assay Kit II (Bio-Rad). For the immunoprecipitation analysis, 500 µg of total cell lysates were precleared with protein A plus G-Sepharose before incubation with specific antibodies, followed by the addition of protein A plus G-Sepharose. The precipitated proteins were resolved in 2× SDS-PAGE sample buffer and separated by electrophoresis on 10%–12% SDS-PAGE. After transfer onto a polyvinylidene difluoride membrane (Millipore Corp.), they were incubated with anti-JAK1 or JAK2 or an anti-Flag antibody and further incubated with a horseradish peroxidase-conjugated secondary antibody (Santa Cruz).

### Cytokine Detection

The cytokine protein analysis experiments were performed by homogenization of relevant tissues in PBS + 1% (*v*/*v*) Triton X cytokines were assayed using the mouse cytokine array panel kit (ary006; R&D Systems) according to the manufacturer’s instructions.

### Tissue Immunohistochemistry

The spinal cord segments were harvested, post-fixed, and sectioned. The sections were incubated with polyclonal rabbit anti-gecko SOCS3 or mouse anti-SOCS3 antibodies (1:500 dilution, Abcam), monoclonal mouse anti-CD68 antibody (1:200, Abcam), monoclonal mouse anti-OX42 antibody (1:200, Abcam), or mouse anti-NeuN antibody (1:200, Abcam) at 4 °C for 36 h. The sections were further reacted with the Cy3-labeled goat anti-mouse IgG secondary antibody (1:400, Gibco) or the FITC-labelled goat anti-rabbit IgG secondary antibody (1:400, Gibco) at 4 °C overnight, followed by observation under a confocal laser scanning microscope (Leica, Heidelberg, Germany).

### Statistical Analysis

The statistical significance of differences between groups was analyzed by one-way analysis of variance (ANOVA) followed by Bonferroni’s *post-hoc* comparison tests with SPSS 15.0 (SPSS, Chicago, IL). The normality and homoscedasticity of the data were verified before any statistical analysis using Levene’s test. Statistical significance was set at the *P* < 0.05 level.

## Results

### GM-CSF and IFN-γ are Strongly Induced After SCI Without Evoking an Excessive Inflammatory Reaction

**Fig. 1:**
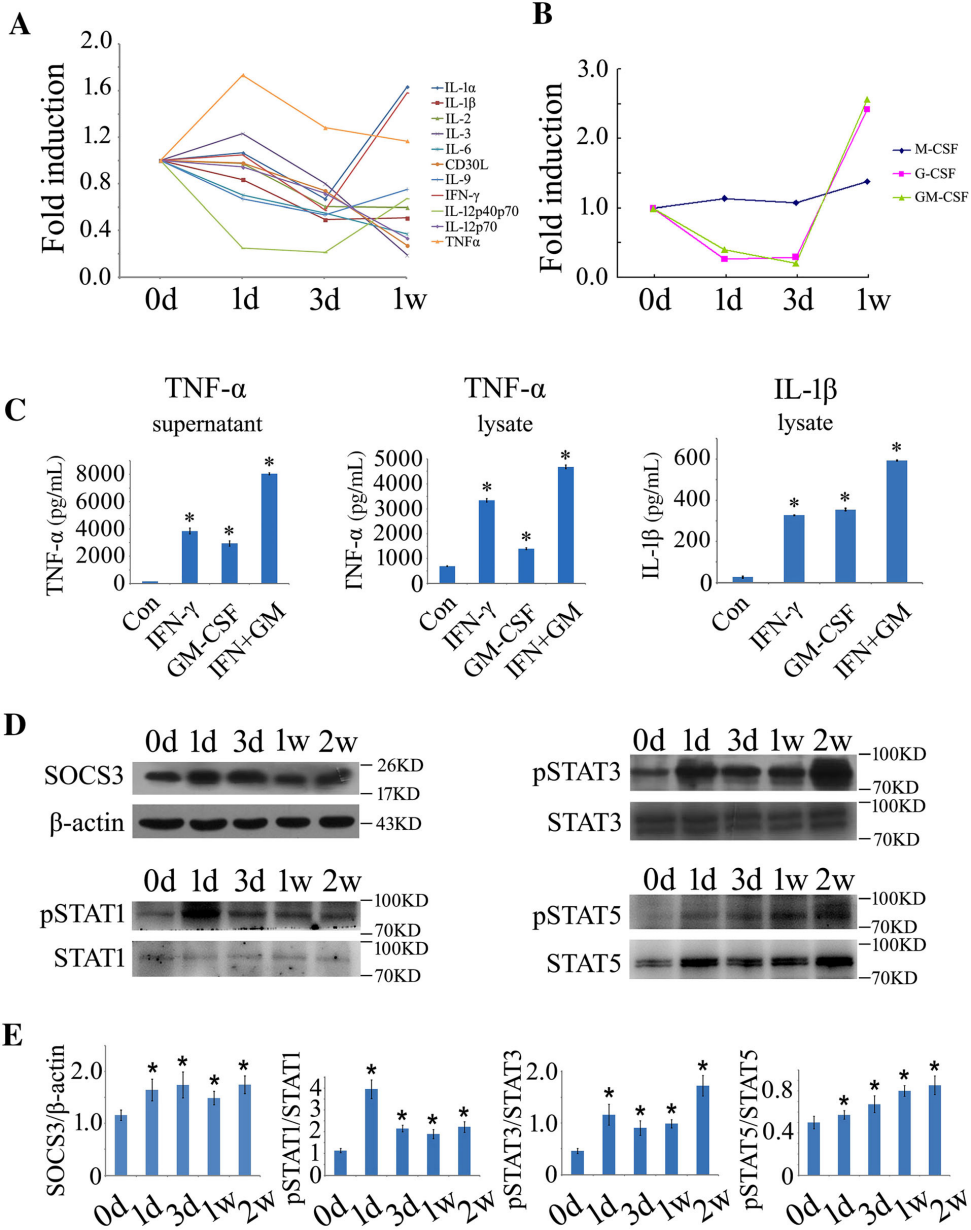
Determination of inflammatory cytokines and gSOCS3 protein levels in the injured spinal cord of gecko and cell cultures. **A**, **B** Inflammatory cytokine profiles of 0.5-cm cord segments at the lesion sites following gecko tail amputation at 0, 1, and 3 days (d) and 1 week (w). Each data point shows the mean of two separate experiments with 10 animals/sample. **C** Production of TNF-α and IL-1β assessed by ELISA after RAW 264.7 cells were stimulated with recombinant GM-CSF (50 ng/mL) and/or IFN-γ (10 ng/mL) for 2 h. **D** Western blots of gSOCS3 and STAT1/3/5 phosphorylation in 0.5-cm segments of injured spinal cord at different times following gecko tail amputation (*n* = 6). **E** Statistical analysis of data as in (**D**). Data are expressed as the mean ± SEM; *P < 0.05.

Mammalian SCI always elicits a robust inflammatory response through the activation of microglia/macrophages by DAMPs or inflammatory cytokines [39, 40]. To understand whether SCI is able to induce equivalent inflammatory profiles in a regenerating model, we screened 0.5-cm cord segments at the lesion sites following gecko tail amputation with a mouse cytokine protein detection array that has been shown to be effective even in amphibians [39]. Tests of several typical cytokines in the amniotic gecko also show consistent results [31]. A total of 40 cytokines, chemokines, and inflammatory markers have been examined (data partially shown). Unexpectedly, we detected low levels of pro-inflammatory cytokine profiles that are normally induced in the mammalian counterparts. TNF-α expression was moderately elevated at 1 day but decreased with the progression of regeneration. The expression of IFN-γ, together with IL-1α, was induced from 3 days after cord amputation. In contrast, the production of other detected cytokines was suppressed in the regenerative process (Fig. 1A).

The myeloid colony-stimulating factors that are relevant to proliferation, differentiation, polarity, and secretion of the cytokines of myeloid cells were also simultaneously determined. M-CSF and G-CSF are important for steady-state myelopoiesis, while GM-CSF is becoming increasingly known as a major mediator of tissue inflammation [41]. Both GM-CSF and G-CSF were significantly induced from 3 days after cord amputation, whereas M-CSF expression showed a slight elevation (Fig. 1B). Dissimilar to SCI in mammals, SCI in the gecko does not evoke excessive inflammation, even though the expression of GM-CSF and IFN-γ is aberrantly induced.

To evaluate the effects of both GM-CSF and IFN-γ on the activation of inflammatory responses, macrophage RAW 264.7 cells were stimulated with recombinant GM-CSF (50 ng/mL) and/or IFN-γ (10 ng/mL) proteins for 2 h. These two cytokines were highly efficient in inducing TNF-α and IL-1β production in the macrophages, although there were no effects on the secretion of IL-1β. IFN-γ showed a synergistic effect on the activation of inflammation (Fig. 1C).

### Expression of gSOCS3 was Dynamically Induced in the Microglia/Macrophages of the Regenerating Spinal Cord Following SCI

Since GM-CSF and/or IFN-γ are insufficient to activate the inflammatory response in the injured spinal cord of the regenerative model, contrary to reports *in vitro*, gecko SOCS3 (gSOCS3) was thought to function in the negative regulation of the GM-CSF- and IFN-γ-mediated signaling pathways. To test this possibility, the protein levels of gSOCS3 were first examined in the 0.5-cm cord segments proximal to the amputation plane. Western blot analysis demonstrated that the expression of gSOCS3 in the injured spinal cord specimens collected at 0, 1, and 3 days, and 1 and 2 weeks increased from 1 day onwards and was sustained to 2 weeks (Fig. 1D, E). Accordingly, its upstream regulators STAT1, STAT3, and STAT5 were also dynamically activated through phosphorylation (Fig. 1D, E). These data indicate that the expression of gSOCS3 is dynamically induced by SCI following tail amputation in the gecko.

**Fig. 2:**
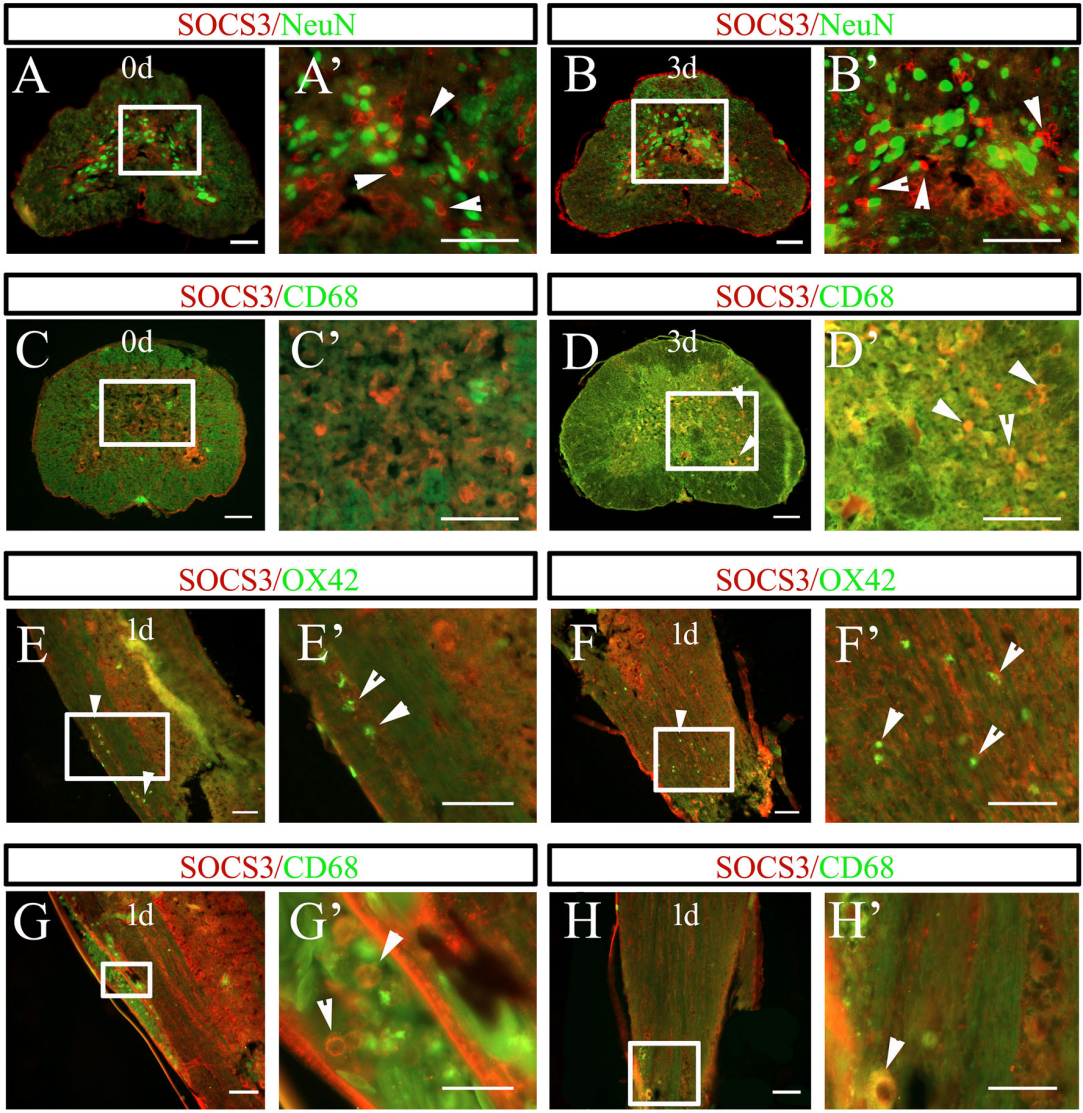
Tissue distribution of gSOCS3 in the injured spinal cord following tail amputation (*n* = 5). **A**–**D** Representative images showing the absence of gSOCS3 in NeuN-positive neurons, but co-localization with CD68-positive microglia/macrophages at 0 and 3 days (rectangles, regions magnified in **A’**–**D’**). **E**–**H** Representative images showing co-localization of gSOCS3 with CD68- but not with OX42-positive cells in the injured spinal cord 1 day after tail amputation (rectangles, regions magnified in **E**’–**H**’). Arrowheads indicate positive staining. Scale bars, 100 μm in **A**–**D** and **A’**–**D’**; 50 μm in **E**–**H**; 25 μm in **E’**–**H’**.

In mammals, SOCS3 has been shown to negatively regulate axonal growth and inflammatory activation [24, 29, 42–44]. To better understand the roles of gSOCS3 in the regenerating spinal cord, we observed its localization in the microglia/macrophages and neurons of the amputated spinal cord in the gecko. Immunostaining showed that gSOCS3 was absent from NeuN-positive neurons but was co-localized with CD68-positive macrophages at 3 days following amputation (Fig. 2A–D). The gSOCS3 protein was undetectable in the resident microglia when they were immunostained with OX42 but was expressed by the infiltrating macrophages in both vessels and lesioned cord on days 1–3 after tail amputation (Fig. 2D–H). The data indicate that injury-induced gSOCS3 uniquely performs inflammation-related functions in the regenerating spinal cord.

### SOCS3 Orthologs Show High Degrees of Identity in Amniotes

**Fig. 3:**
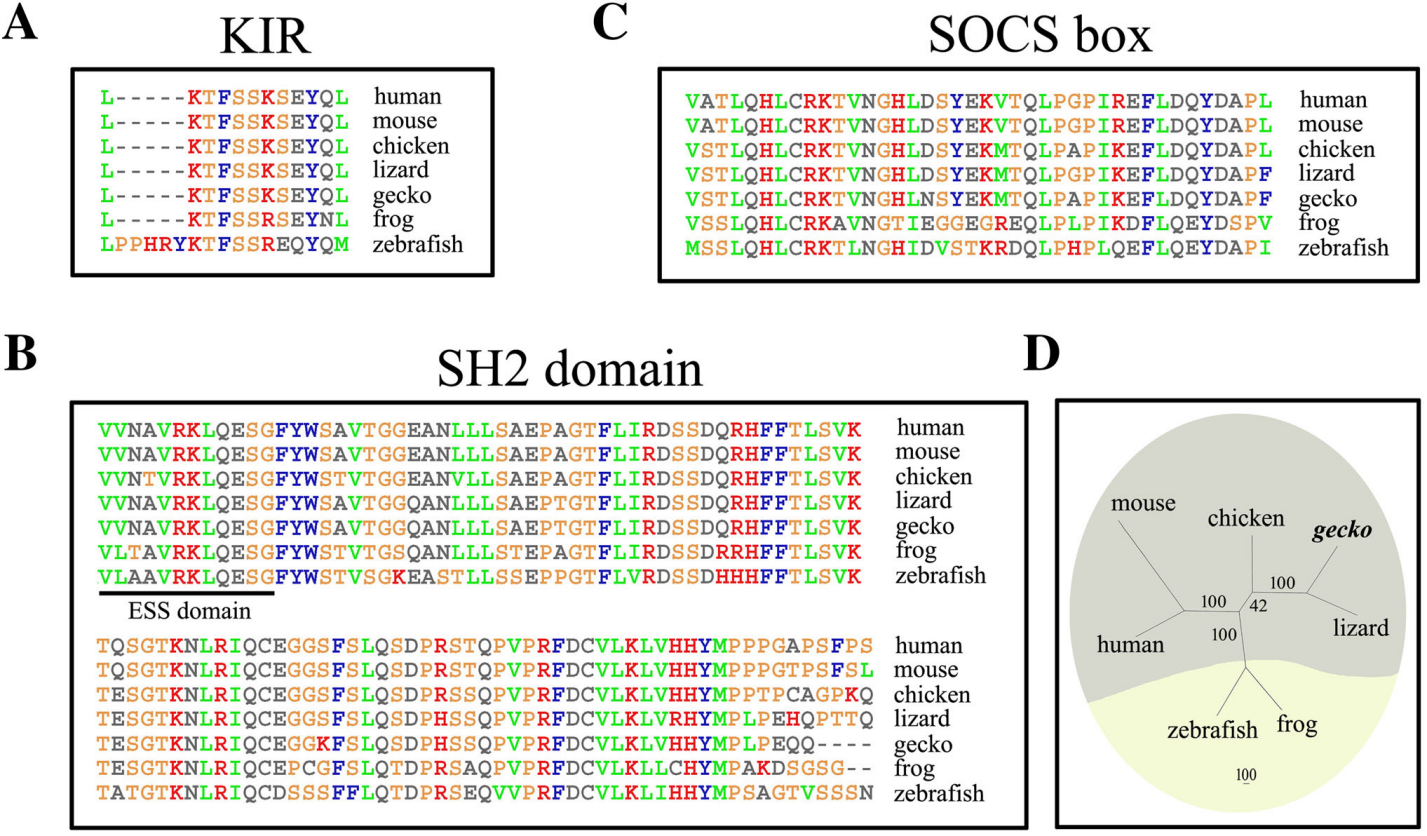
Sequence analysis of gSOCS3. **A**–**C** Sequence alignment of SOCS3 proteins of vertebrates. Gaps introduced into sequences to optimize alignment are represented by dashes. The kinase inhibitory region (KIR) domain (**A**), a central Src-homology 2 (SH2) domain (**B**), and a C-terminal SOCS box (**C**). The 12-amino-acid extended SH2 subdomain (ESS) is underlined. **D** Unrooted phylogenetic tree of SOCS3 proteins from the representative species constructed by the neighbor-joining method in the PHYLIP 3.5c package. Bootstrap majority consensus values on 1000 replicates are indicated at each branch point as percentages. The sequences from GenBank are gecko *Gekko japonicus* SOCS3 (XP_015272482); human *Homo sapiens* SOCS3 (NP_003946); mouse *Mus musculus* SOCS3 (NP_031733); chicken *Gallus gallus* SOCS3 (NP_989931); green anole *Anolis carolinensis* SOCS3 (XP_008102404); frog *Xenopus tropicalis* SOCS3 (NP_001005696); and zebrafish *Danio rerio* SOCS3 (NP_998469).

To shed light on the structural characteristics of gSOCS3, we cloned *gSOCS3* (GenBank accession number XP_015272482) and aligned the deduced amino-acid sequence with those of other vertebrates. Results showed that gSOCS3 consists of a KIR domain, SOCS (suppressor of cytokine signaling) box, and SH2 (Src-homology 2) domain [45, 46], sharing 88.8%, 89.8%, 90.2%, and 96.1% identity with human, mouse, chicken and lizard SOCS3, respectively, while it shares only 64.2% identity with zebrafish and 74.9% with frog (Fig. 3A–C). The phylogenetic tree showed that gSOCS3 clusters with those of amniotes rather than with regenerative model organisms (Fig. 3D), suggesting a conserved physiological function of SOCS3 during the evolution of amniotes.

### Enforced Expression of gSOCS3 Attenuates the Production of Inflammatory Cytokines Mediated by GM-CSF/IFN-γ

**Fig. 4:**
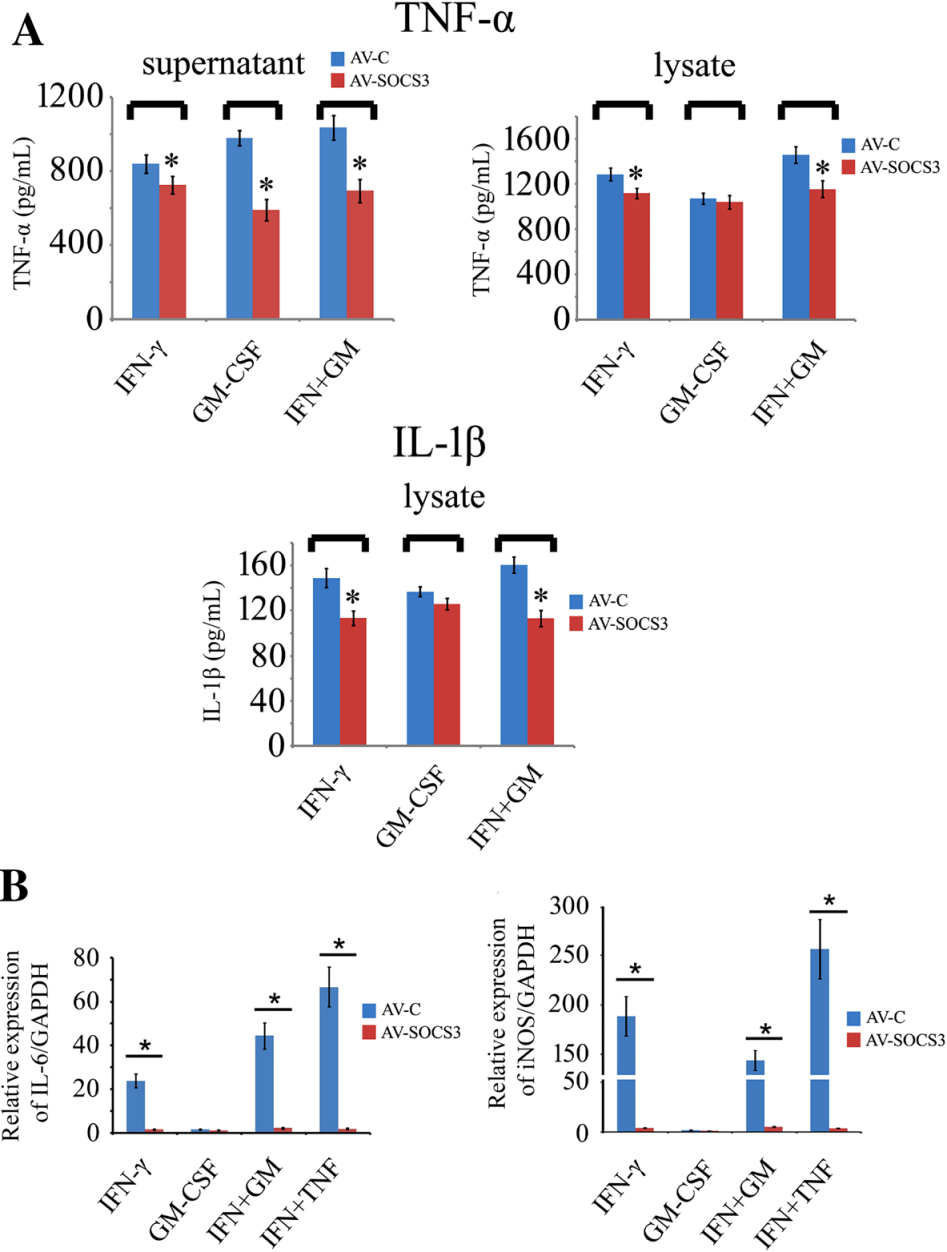
Enforced expression of gSOCS3 reduces the production of inflammatory cytokines mediated by GM-CSF/IFN-γ. **A** TNF-α and IL-1β protein levels determined by ELISA in supernatants and lysates of cell cultures. RAW 264.7 cells were transfected with GV314-SOCS3 or GV314-vector adenovirus for 48 h following treatment with recombinant GM-CSF (50 ng/mL) or/and IFN-γ (10 ng/mL) protein for 2 h. **B** Expression of IL-6 and iNOS assessed by RT-PCR after cell treatment with the same approach as in **A**. Data are expressed as the mean ± SEM; *P < 0.05.

To investigate the negative regulatory action of gSOCS3 in the GM-CSF/IFN-γ-mediated inflammatory response, macrophage RAW 264.7 cells were transfected with GV314-SOCS3 or GV314-vector adenovirus for 48 h, followed by treatment with recombinant GM-CSF (50 ng/mL) or/and IFN-γ (10 ng/mL) protein for 2 h. ELISA assays revealed that the enforced expression of gSOCS3 was able to significantly decrease the levels of TNF-α and IL-1β in the supernatant and/or lysate (Fig. 4A). Furthermore, it suppressed the expression of other inflammatory cytokines, such as IL-6 and iNOS (Fig. 4B). These data indicate that gSOCS3 overexpression efficiently attenuates the production of inflammatory cytokines that is mediated by GM-CSF/IFN-γ.

### gSOCS3 Inhibits the JAK/STAT Pathway that is Triggered by GM-CSF/IFN-γ

**Fig. 5:**
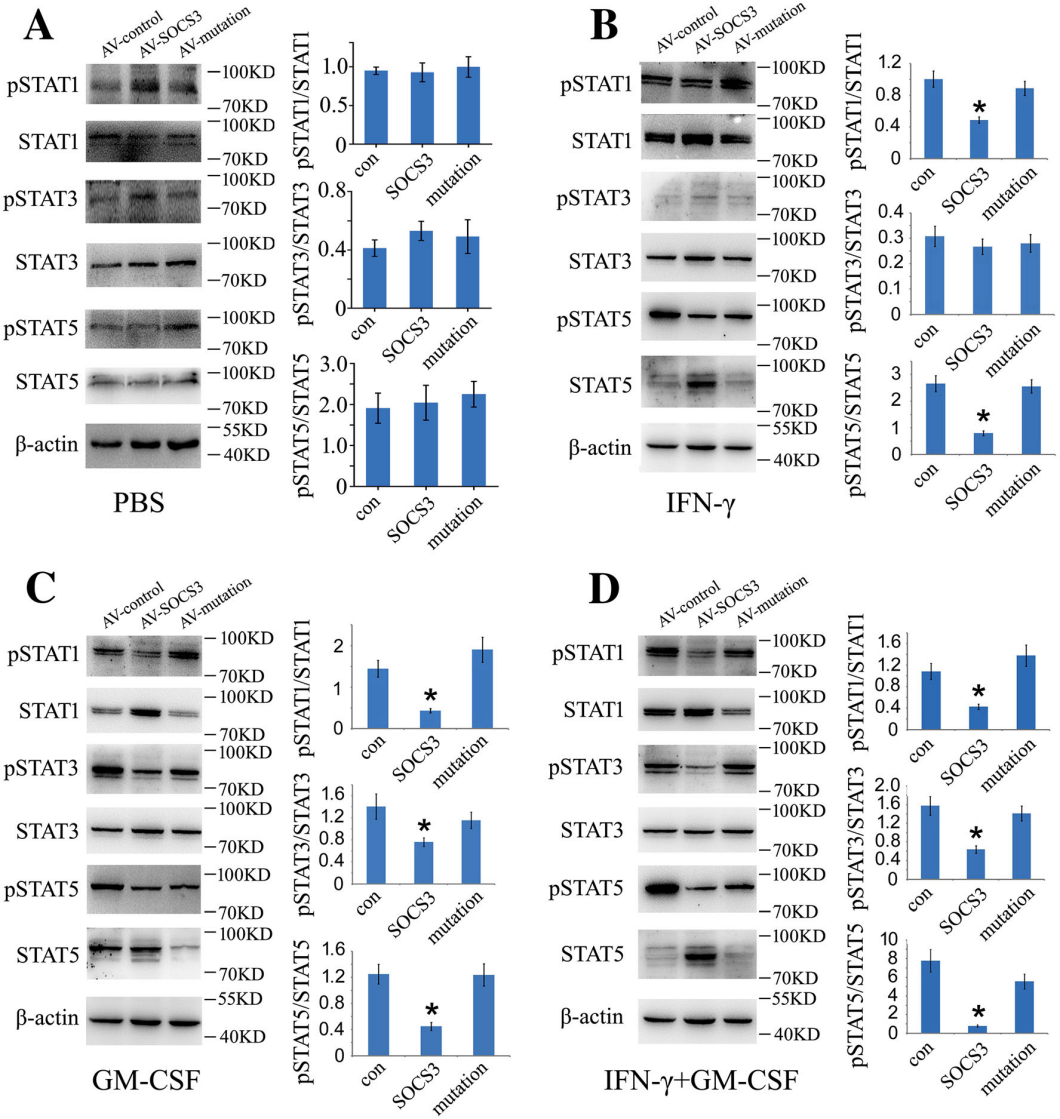
gSOCS3 negatively regulates the phosphorylation of STAT1/3/5 through the KIR domain. **A**–**D** Macrophages were transfected with GV314-SOCS3 or GV314-mSOCS3 (F4A in KIR domain) adenovirus for 48 h before treatment with 0.1 mol/L PBS (pH 7.4) (**A**), 10 ng/mL IFN-γ (**B**), 50 ng/mL GM-CSF (**C**), or combinations of 50 ng/mL GM-CSF and 10 ng/mL IFN-γ (**D**) for 2 h. Phosphorylation of STAT1/3/5 was determined by Western blot. Data are expressed as the mean ± SEM; *P < 0.05.

The cytokines GM-CSF and IFN-γ are able to activate the JAK/STAT signaling, which appears to govern the inflammatory signature [47–49]. To demonstrate that the suppression of inflammation by gSOCS3 is involved in inhibiting the activation of the STATs, the protein levels of phosphorylated STAT1, STAT3, and STAT5 were determined following macrophage transfection with control, GV314-SOCS3, or GV314-mSOCS3 (F4A in KIR) for 48 h. The cells were additionally incubated with recombinant GM-CSF (50 ng/mL) or/and IFN-γ (10 ng/mL) for 2 h. The results revealed that the enforced expression of gSOCS3 significantly reduced the phosphorylation of STAT1 and STAT5 in comparison with the control following the stimulation of cells with IFN-γ (Fig. 5A, B). By contrast, it decreased the levels of pSTAT1, pSTAT3, and pSTAT5 after the stimulation of cells with GM-CSF or a combination of GM-CSF and IFN-γ (Fig. 5C, D). Notably, gSOCS3 was also efficient in limiting the phosphorylation of STAT1 and STAT3 when cells were treated with TNF-α/IFN-γ (Fig. S1). SOCS3 with a KIR mutation (GV314-mSOCS3) failed to inhibit the JAK/STAT pathway (Fig. 5A–D), suggesting that the KIR domain is indispensable for the inhibitory function of SOCS3.

### gSOCS3 Suppresses GM-CSF/IFN-γ-Mediated Inflammation *via* Negative Regulation of JAK1 and JAK2

**Fig. 6:**
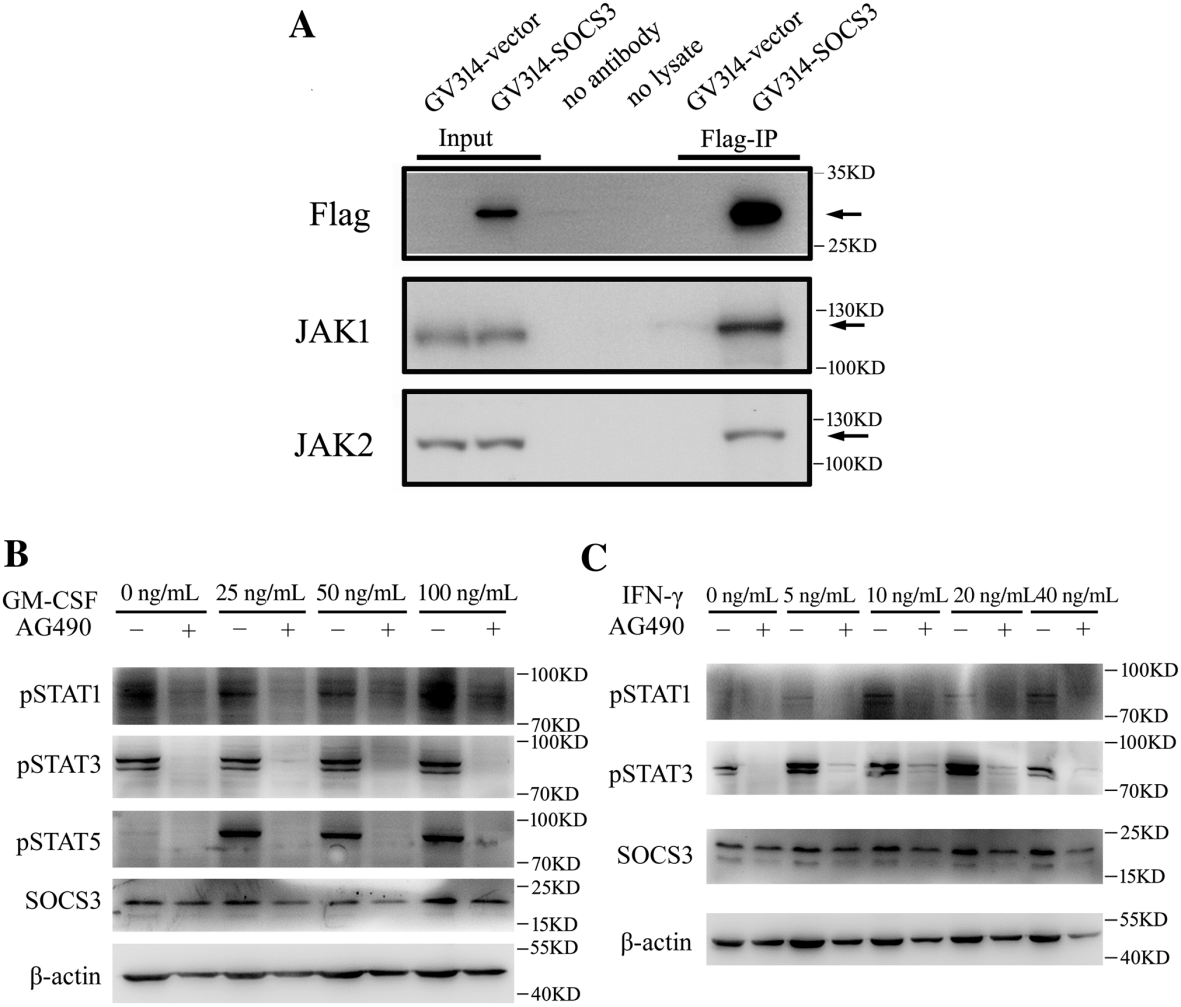
gSOCS3 binds with JAK1 and JAK2 to negatively regulate the activation of STAT1/3/5. **A** Binding assays of gSOCS3 with JAK1/2 in macrophages transfected with GV314-SOCS3 adenovirus for 72 h. Immunoprecipitation using anti-Flag antibody and detection of the components of the Flag(SOCS3)-associated complexes with anti-JAK1 or -JAK2 antibody. **B, C** Addition of 100 μmol/L JAK1/2 inhibitor AG490 decreases the levels of pSTAT1/3/5 in the context of stimulation with the cytokine GM-CSF or IFN-γ.

SOCS3 has been shown to bind to the gp130 receptor or to JAK1 and JAK2 proteins to inhibit the signal transduction induced by IL-6 [27]. Thus, in the context of GM-CSF/IFN-γ stimulation, JAK1 and/or JAK2 are potential target(s) of SOCS3 action. To clarify the kinase(s) that interact with gSOCS3, co-immunoprecipitation was applied to assay the JAKs/gSOCS3 interaction. Macrophage RAW 264.7 cells were transfected with GV314-SOCS3 or GV314-vector for 72 h, followed by protein extraction. As shown in Fig. 6, both JAK1 and JAK2 were present in the Flag-associated complexes when they were immunoprecipitated with the anti-Flag antibody (Fig. 6A). These results indicate that gSOCS3 negatively regulates JAK1/2 following stimulation with GM-CSF/IFN-γ.

To further validate that the activity of JAK1/2 is critical for GM-CSF/IFN-γ-related signal transduction, the JAK1/2-specific inhibitor AG490 was added at a concentration of 100 μmol/L to the culture medium of the macrophages that also contained different concentrations of recombinant GM-CSF or IFN-γ. The results showed that the addition of AG490 inhibited the downstream activation of STAT1, STAT3, and STAT5, as well as downstream SOCS3, which were significantly induced by recombinant GM-CSF at 0 ng/mL–100 ng/mL or IFN-γ at 0 ng/mL–40 ng/mL (Fig. 6B, C).

### SOCS3 is Linked to GM-CSF/IFN-γ-Induced Inflammatory Tolerance

**Fig. 7:**
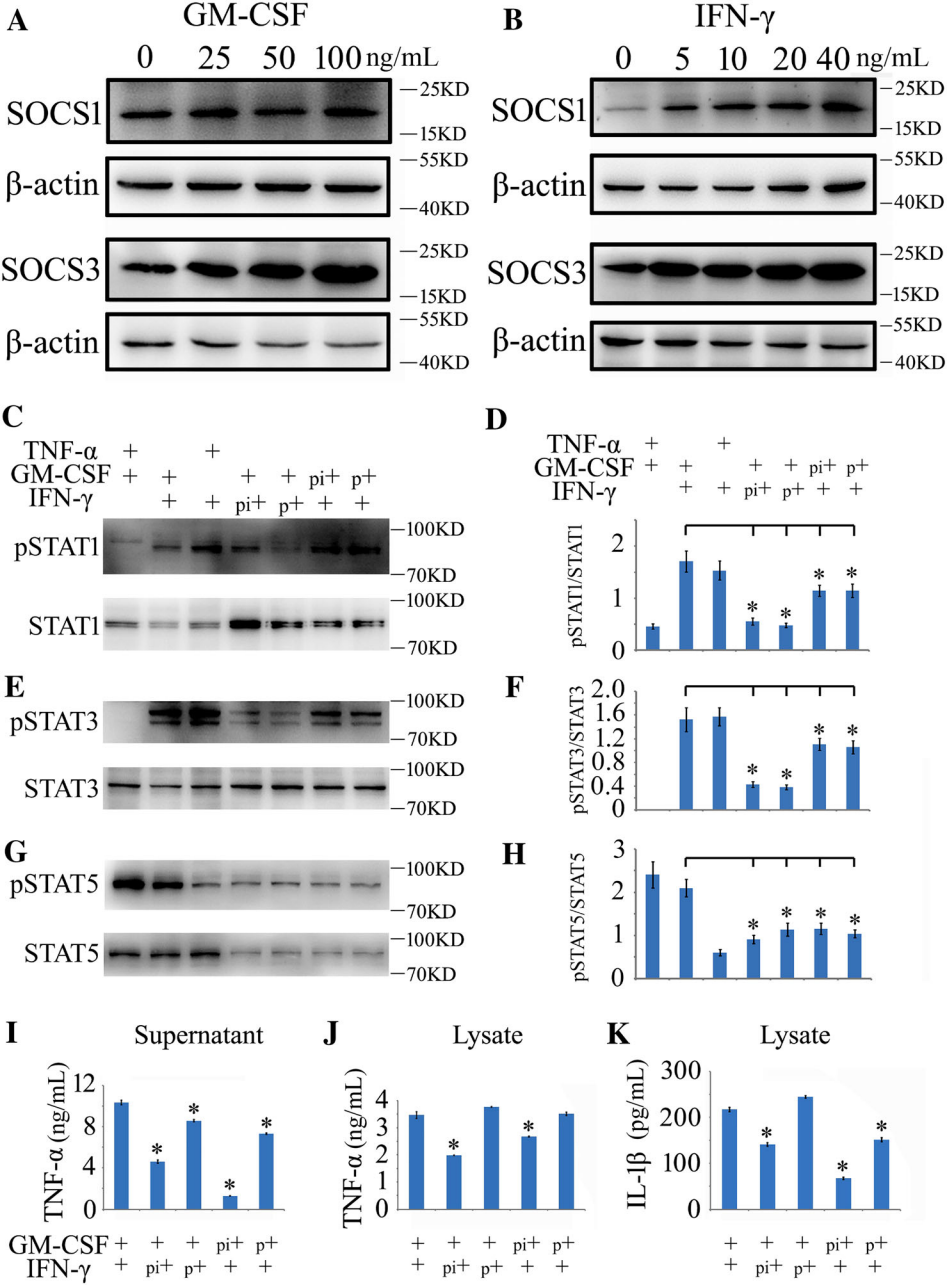
Determination of GM-CSF/IFN-γ-induced inflammatory tolerance mediated by SOCS3. **A**, **B** Western blots showing that treatment with IFN-γ or GM-CSF recombinant protein induces the expression of endogenous SOCS1 and/or SOCS3 in macrophages following treatment at different concentrations for 0.5 h. **C**, **E**, **G** Western blots of pSTAT1/3/5 following different treatments. pi indicates GM-CSF or IFN-γ pretreatment for 0.5 h without a change of culture medium, followed by the addition of the other cytokine for 1.5 h; p indicates pretreatment with GM-CSF or IFN-γ for 0.5 h with a change of medium, followed by the other cytokine for 1.5 h. **D**, **F**, **H** Statistical analysis of data as in **C**, **E**, and **G**, respectively. **I**–**K** ELISA assays of TNF-α and IL-1β in the supernatant and lysate of cell cultures after various treatments. Data are expressed as the mean ± SEM; *P < 0.05.

Different concentrations of GM-CSF induced the expression of endogenous SOCS3 in macrophages, while IFN-γ induced both SOCS1 and SOCS3 after cell incubation for 0.5 h (Fig. 7A, B). An elevation of intracellular SOCS3 that was induced by pre-stimulation with one cytokine likely desensitized the activation of the JAK/STAT pathway by the other cytokines. To address the correlations, macrophages were pretreated with recombinant IFN-γ (10 ng/mL) or GM-CSF (50 ng/mL) protein for 0.5 h, followed by a secondary stimulation with recombinant GM-CSF (50 ng/mL) or IFN-γ (10 ng/mL) for 1.5 h with or without changes of the culture medium. Combinations of IFN-γ/GM-CSF, GM-CSF/TNF-α (10 ng/mL), or TNF-α (10 ng/mL)/IFN-γ were simultaneously performed as controls. The results demonstrated that the pretreatment of macrophages with GM-CSF or IFN-γ decreased the levels of pSTAT1, pSTAT3, and pSTAT5 in response to a secondary challenge with the other (Fig. 7C–H). Accordingly, the subsequent production of TNF-α and IL-1β was also suppressed by such pretreatment (Fig. 7I–K). These data indicate that SOCS3 is involved in GM-CSF/IFN-γ-induced inflammatory tolerance.

### Overexpression of gSOCS3 in the Severed Spinal Cord Decreases the Levels of Inflammatory Cytokines

**Fig. 8:**
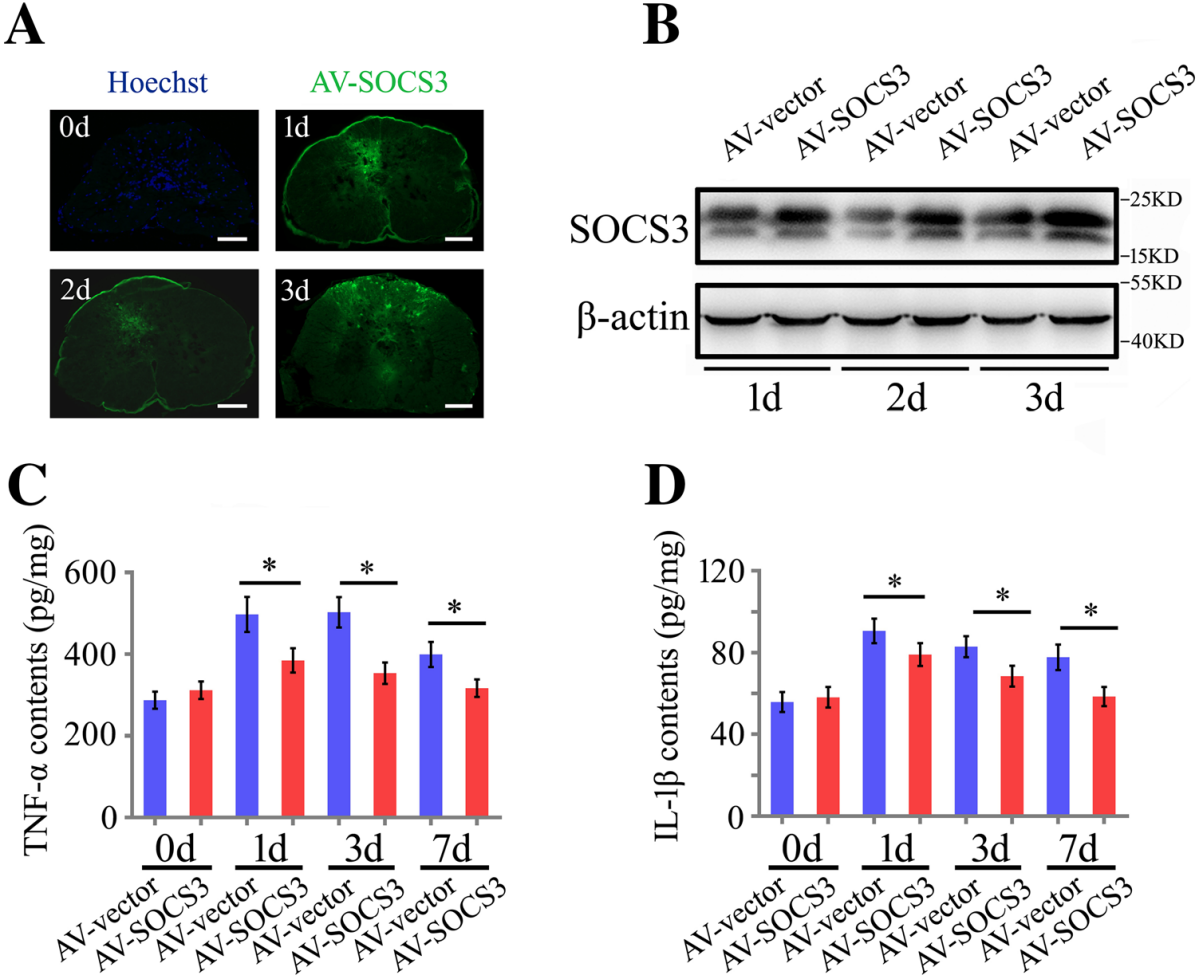
gSOCS3 overexpression in the injured spinal cord decreases the levels of inflammatory cytokines. **A** Representative images of GFP fluorescence in the injured cord at 1, 2, and 3 days (scale bars, 100 μm). **B** Western blots of gSOCS3 in the injured spinal cord following transfection with GV314-SOCS3 adenovirus. **C**, **D** ELISA assays of TNF-α and IL-1β production in the injured spinal cord transfected with GV314-SOCS3 adenovirus on days 0, 1, 3 and 7. Data are expressed as the mean ± SEM; *P < 0.05.

To elucidate the physiological function of gSOCS3 in the injured spinal cord, a total of 9 μL of GV314-SOCS3 or GV314-vector adenovirus in three aliquots were immediately injected into the intervertebral space 2 mm–3 mm anterior to the amputation plane of the gecko tail. The transfection efficiency was evaluated by the intensity of GFP fluorescence in cross-sections of the spinal cord at 1, 2, and 3 days following adenovirus injection (Fig. 8A). Western blot analysis also showed a significant elevation of gSOCS3 expression in the cord, indicating efficient transfection in this non-mammalian species (Fig. 8B). Notably, the adenovirus was found to infect not only the macrophages/microglia but other cell types in the cord (data not shown). The ELISA data demonstrated that the production of TNF-α and IL-1β was significantly reduced following GV314-SOCS3 transfection at 1, 3, and 7 days (Fig. 8C, D), emphasizing the important roles of gSOCS3 in blocking the inflammatory response in the injured spinal cord.

## Discussion

SOCS3 is intracellularly induced by multiple cytokines in a variety of cell types, such as myeloid-derived cells and nerve cells, in response to stimulation with TNF-α, IL-1, lipopolysaccharide (LPS), ciliary neurotrophic factor, oncostatin M, insulin-like growth factor-1, and IL-6 [24, 27]. In the mammalian central and peripheral nervous systems, SOCS3 is upregulated in neuronal and glial cells, inhibiting neuronal protection and axonal regeneration, as well as decreasing astrocytic reactivity through negative regulation by STAT3 phosphorylation [26, 29, 43, 44, 50–52]. SOCS3 is also inducibly expressed in dendritic cells, T cells, and macrophages, which are recruited to the CNS under inflammatory conditions [24]. Interestingly, SOCS3 is absent from the neurons of the gecko spinal cord, suggesting its important roles in suppressing inflammation, rather than in affecting axonal regrowth. Whether such a strategy is used by other models of regeneration remains unclear. By screening the gecko genome, we found two paralogs of SOCS3, designated SOCS3a and SOCS3b (Fig. S2A). In the present study, gSOCS3 clustered with and was thus named SOCS3b. SOCS3a, which lacks a KIR domain in the N-terminus, co-localized with neurons (Fig. S2B, C). Further investigation will be helpful in elucidating the distinct functions of the two paralogs in animal models of regeneration.

GM-CSF, together with other CSFs, was first regarded as a pro-inflammatory cytokine due to its ability to stimulate the formation of the neutral protease plasminogen activator in macrophages [53]. This pleiotropic cytokine is secreted by a wide variety of cells, including endothelial cells, monocytes, astrocytes, and T cells [54]. Aberrant expression of GM-CSF is associated with multiple neurological disorders such as Alzheimer’s disease (AD), vascular dementia, and multiple sclerosis [55, 56]. Neutralization of GM-CSF by antibodies is capable of suppressing microglial activation in the cerebral cortex of a mouse AD model [57]. Therefore, GM-CSF promotes CNS inflammation *via* microglial activation. Studies on GM-CSF-induced pro-inflammatory mediators have shown different outcomes. For example, GM-CSF fails to induce TNF-α and NO in the activation of microglia [54], while a low dose of recombinant GM-CSF is sufficient to facilitate the production of TNF-α in human mononuclear cells [17]. LPS has been shown to significantly increase the GM-CSF-induced production of cytokines, indicating that GM-CSF might directly or indirectly activate inflammation, depending on the different cell types. In this study, we demonstrated that GM-CSF induced the production of TNF-α and IL-1β by macrophages, with IFN-γ acting in a synergistic manner. GM-CSF-mediated CNS inflammation is re-emphasized following SCI.

GM-CSF activates intracellular JAK2/STAT5 signaling through the GM-CSF receptors of the plasma membrane and is required for the proliferation, differentiation, and inflammatory activation of myeloid cells [10]. Although enforced gSOCS3 inhibited the phosphorylation of STAT1 and STAT3 in macrophages (Fig. 5C), the GM-CSF-mediated inflammatory response appeared to be exclusively regulated *via* STAT5, as the addition of recombinant GM-CSF to macrophage cultures resulted in insignificant increases of pSTAT1 and pSTAT3 (Fig. 6B). In contrast, IFN-γ activated STAT1 and STAT3 through JAK2 signaling (Fig. 6C), consistent with the findings of other investigations [49, 58]. However, gSOCS3 overexpression only reduced the levels of pSTAT1 and pSTAT5 (Fig. 5B), suggesting an unknown mechanism of dephosphorylation in STAT3 resembling that of STAT1 by protein tyrosine phosphatases [59].

Pretreatment of the macrophages with GM-CSF or IFN-γ resulted in desensitization of the JAK/STAT pathway in a secondary cytokine challenge through reductions in pSTAT1, pSTAT3, and pSTAT5. A low concentration of IFN-γ (5 ng/mL) activates SOCS1 and SOCS3 within 20 min [58], and this is sufficient to negatively regulate the activities of JAK1 and JAK2, leading to suppressed phosphorylation of downstream STAT1, STAT3, and STAT5. GM-CSF has also been shown to induce intracellular SOCS3, which might be effective in the desensitization of the JAK/STAT pathway that is mediated by other cytokines.

In conclusion, SOCS3 is specifically induced in the microglia rather than in the neurons of the regenerating spinal cord. This protein acts to limit excessive inflammatory responses in the macrophages/microglia that are mediated by GM-CSF/IFN-γ through the negative regulation of JAK1/2 activity. Our results reveal a distinct regulatory phenomenon of SOCS3 in the injured spinal cord of non-mammalian amniotes.

## Electronic supplementary material

Below is the link to the electronic supplementary material.

Supplementary material 1 (PDF 297 kb)
