# Supplementary material for: SOCS3 Attenuates GM-CSF/IFN-γ-Mediated Inflammation During Spontaneous Spinal Cord Regeneration
Source: Neurosci Bull. 2020 Apr 18;36(7):778–92. doi: 10.1007/s12264-020-00493-8 (PMC7340708; doi:10.1007/s12264-020-00493-8)
Supplement: Supplementary file 1 — Supplementary material 1 (PDF 297 kb) [file 12264_2020_493_MOESM1_ESM.pdf]

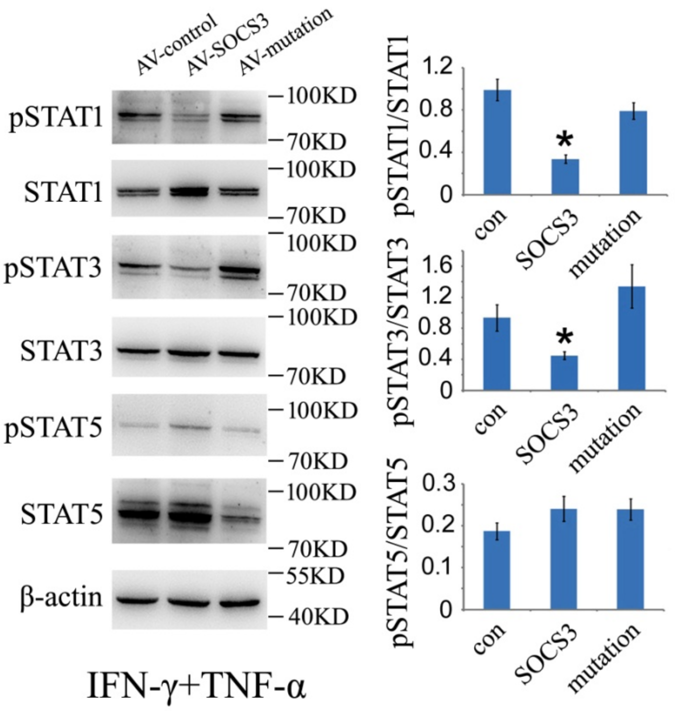

Fig. S1 gSOCS3 negatively regulates the phosphorylation of STAT1/3/5 through the KIR domain. Macrophages were transfected with GV314-SOCS3 or GV314-mSOCS3 (F4A in KIR domain) adenovirus for 48 h before treatment with combinations of 10 ng/mL IFN-γ and 10 ng/mL TNF-α for 2 h. Phosphorylation of STAT1/3/5 was determined by Western blot. Data are expressed as the mean ± SEM; *$p < 0.05$.

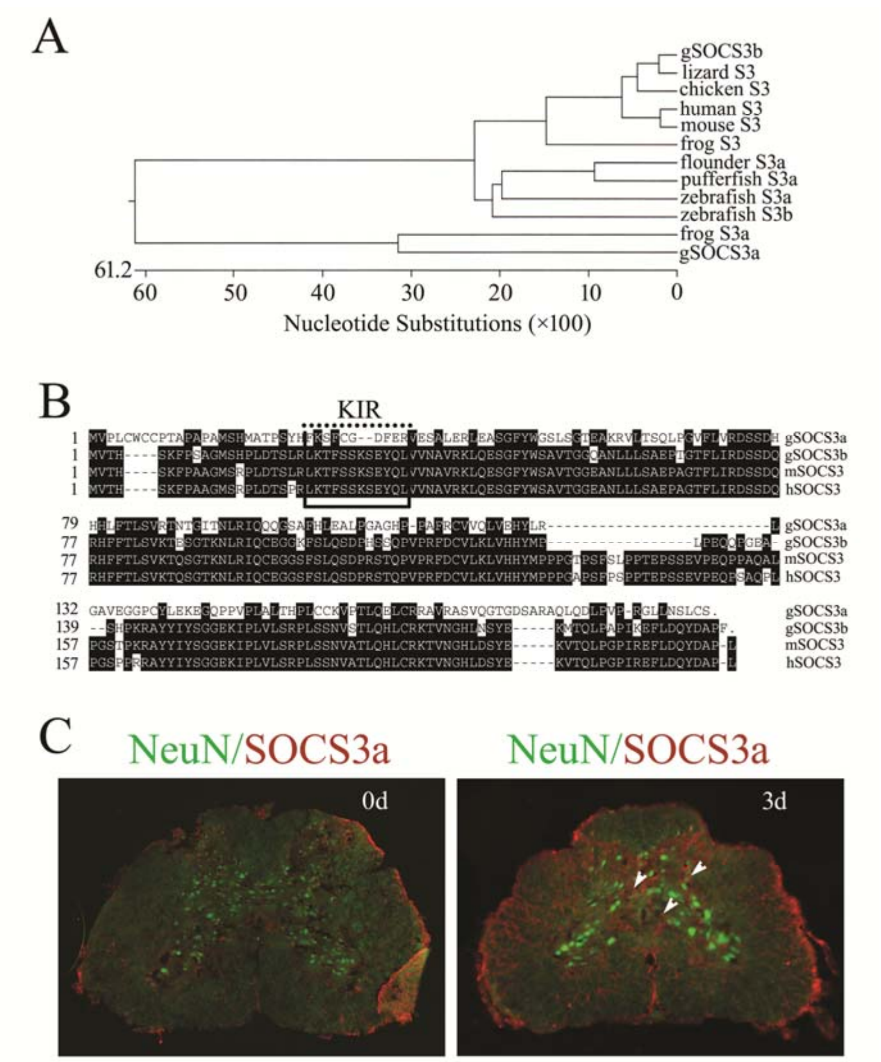

Fig. S2 Analysis of two subtypes of gecko SOCS3 and the distribution of gSOCS3a in the neurons of injured gecko spinal cord. **A** Phylogenetic tree of SOCS3 proteins from representative species constructed by the neighbor-joining method in the PHYLIP 3.5c package. The sequences from GenBank are gecko *Gekko japonicus* SOCS3a (XP_015269902); flounder *Paralichthys olivaceus* SOCS3a (BAW03226); pufferfish *Tetraodon nigroviridis* SOCS3a (ABC60040); frog *Xenopus laevis* SOCS3a (XP_018081848); and zebrafish *Danio rerio* SOCS3a (NP_956244). For other sequences, refer to Fig. 3. **B** Sequence alignment of gecko, mouse, and human SOCS3

proteins. C Representative images of the distribution of gSOCS3a in the neurons of injured gecko spinal cord at 0 and 3 days after tail amputation. Arrowheads indicate positive signals.
